# Merkel Cell Carcinoma Masquerading as Dermatitis

**DOI:** 10.7759/cureus.116561

**Published:** 2026-09-19

**Authors:** Marlena Freeman, Gregory Gates, Iris Ahronowitz

**Affiliations:** 1 Dermatology, David Geffen School of Medicine, University of California at Los Angeles (UCLA), Los Angeles, USA; 2 Dermatopathology, David Geffen School of Medicine, University of California at Los Angeles (UCLA), Los Angeles, USA

**Keywords:** atypical merkel cell carcinoma, atypical skin cancer, dermatitis mimicker, merkel cell carcinoma, skin cancer in older adults

## Abstract

Merkel cell carcinoma (MCC) is a rare, highly aggressive neuroendocrine skin carcinoma that typically presents as a rapidly enlarging, firm, painless nodule on sun-exposed areas in elderly patients. This malignancy is characterized by high rates of local recurrence, regional spread, and distant metastasis. We report a case of a woman in her 80s who presented with an erythematous patch on the left anterior thigh, a non-sun-exposed site, initially diagnosed as inflammatory dermatitis and treated with topical corticosteroids. After two months of failed therapy and interval growth, a punch biopsy was performed. Histopathology revealed a dermal infiltrate of small to medium-sized cells with “salt and pepper” chromatin and numerous mitotic figures. Immunohistochemistry confirmed a diagnosis of MCC. Staging with positron emission tomography/computed tomography (PET/CT) revealed an incidental pancreatic lesion with benign cytology and subsequent imaging stability but no evidence of metastatic disease. The staging workup was complicated by acute pancreatitis for which she was hospitalized, and during which she developed new-onset atrial fibrillation and congestive heart failure. After discharge, the patient underwent wide local excision; however, sentinel lymph node biopsy was deferred due to the patient’s acute comorbidities. In conjunction with the patient, the decision was made for close surveillance rather than adjuvant radiation or systemic therapy. At the most recent follow-up, 16 months after excision, there was no evidence of recurrence. This case highlights an atypical presentation of MCC as a dermatitic-appearing patch in a non-sun-exposed area and underscores the importance of biopsy in atypical lesions that do not respond to treatment, as well as the need to individualize treatment in elderly patients with significant competing comorbidities.

## Introduction

Merkel cell carcinoma (MCC) is a rare, aggressively malignant, neuroendocrine-derived cutaneous neoplasm that primarily affects older populations [1-3]. Clinically, MCC most commonly presents on sun-damaged skin of the head, neck, and arms as an erythematous-to-violaceous, indurated plaque or a rapidly enlarging, firm, painless nodule [3,4]. Although rare, MCC arising on non-sun-exposed skin has been documented and, though one case series showed a higher frequency of nodal involvement at diagnosis in such cases, survival data are conflicting and may be more dependent on factors other than sun exposure, including initial tumor size [5-7]. The general aggressive nature of this tumor is underscored by its high rates of local recurrence and nodal metastasis, presenting significant challenges in clinical management [3,8]. Five-year survival is about 51% for localized disease, dropping to as low as 14% with distant metastasis [8]. Given these stakes and MCC's potential to mimic other benign or malignant skin conditions, prompt skin biopsy is critical to guide timely intervention [9].

## Case presentation

A woman in her 80s without prior skin cancer history presented to the dermatology clinic for a routine skin check and noted a persistent asymptomatic pink patch on her left thigh that had appeared approximately two months prior. She reported limited daily photoprotection with SPF 15 in her facial makeup. Her family history was positive for a brother who died of metastatic lung cancer, and a son with a history of basal and squamous cell carcinoma.

On examination, the left anterior thigh demonstrated a 1 cm well-demarcated, erythematous patch without scale and with no associated induration or nodularity. There was no palpable lymphadenopathy. The left thigh lesion was initially thought to be suggestive of an inflammatory dermatitis. The patient was prescribed a trial of topical triamcinolone 0.1% ointment to be applied twice daily. She returned to the clinic two months later. The erythematous patch had not responded to the topical corticosteroid and had expanded to 1.8 cm in diameter on the epidermal surface, though it was still non-palpable (Figure 1).

**Figure 1:**
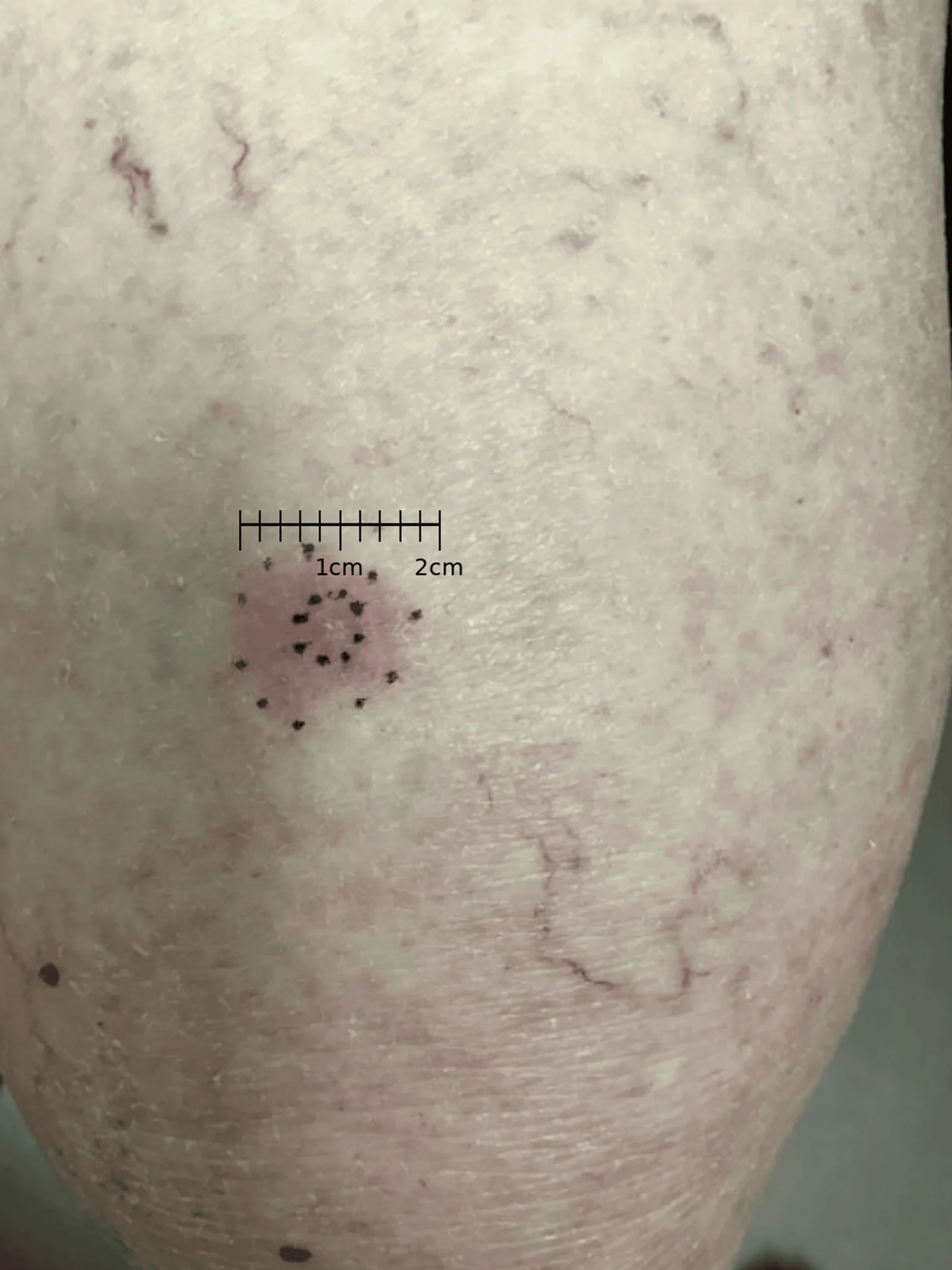
Erythematous patch on the left anterior thigh at the time of biopsy. The patch had increased in size after an unsuccessful trial of triamcinolone 0.1% ointment.

Given the failure to respond to topical steroids, and to rule out cutaneous B-cell lymphoma or other neoplasms, a 4 mm punch biopsy of the left thigh lesion was performed. Histologic sections revealed a small round blue cell tumor diffusely infiltrating the dermis (Figure 2A). The cells formed small-to-medium-sized nests characterized by finely stippled “salt and pepper” chromatin, nuclear pleomorphism, and numerous mitotic figures (Figure 2B). Immunohistochemistry demonstrated that the tumor cells were positive for pan-keratin, CAM5.2, INSM1, and exhibited characteristic perinuclear dot-like staining for cytokeratin 20 (CK20) (Figure 2C). Stains for TTF-1, SOX-10, S100, calcitonin, and CEA were negative. These findings collectively supported the diagnosis of Merkel cell carcinoma (MCC) [3].​

**Figure 2:**
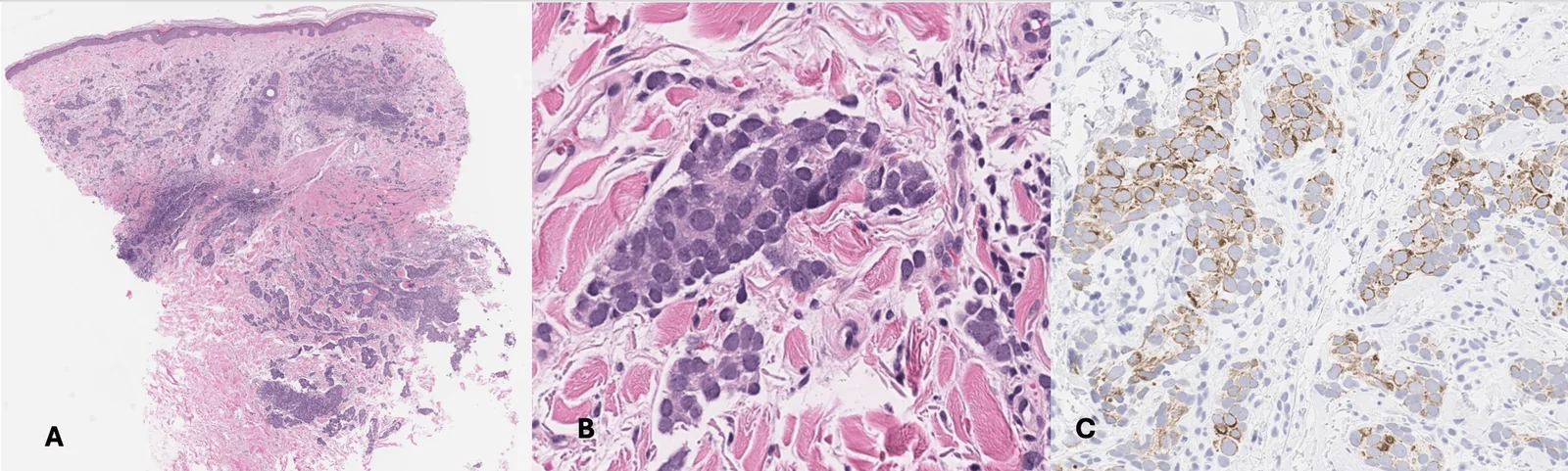
Histopathology of punch biopsy of the skin. (A) Hematoxylin and eosin (H&E) stain, 2x: punch biopsy demonstrating a small round blue cell tumor diffusely infiltrating the dermis. (B) H&E stain, 10x: tumor cells demonstrate finely stippled “salt and pepper” chromatin characteristic of neuroendocrine differentiation. (C) Cytokeratin 20 (CK20), 20x: characteristic paranuclear dot-like positivity, supporting the diagnosis of Merkel cell carcinoma in the appropriate clinical and immunohistochemical context.

The patient underwent a staging workup which was complicated by several acute medical issues. A positron emission tomography/computed tomography (PET/CT) scan revealed no evidence of lymph node or systemic involvement of MCC, and an incidental mixed solid/cystic partially calcified mass at the pancreatic head/uncinate process (Figures 3A-3F). Endoscopic ultrasound-guided fine-needle aspiration and core biopsies were performed, showing a pancreatic lesion with benign cytology. Following pancreatic biopsy, the patient was hospitalized for acute pancreatitis. Her hospitalization was further complicated by new-onset atrial fibrillation and congestive heart failure with restrictive physiology of unclear etiology. She was eventually successfully stabilized on apixaban and furosemide.

**Figure 3:**
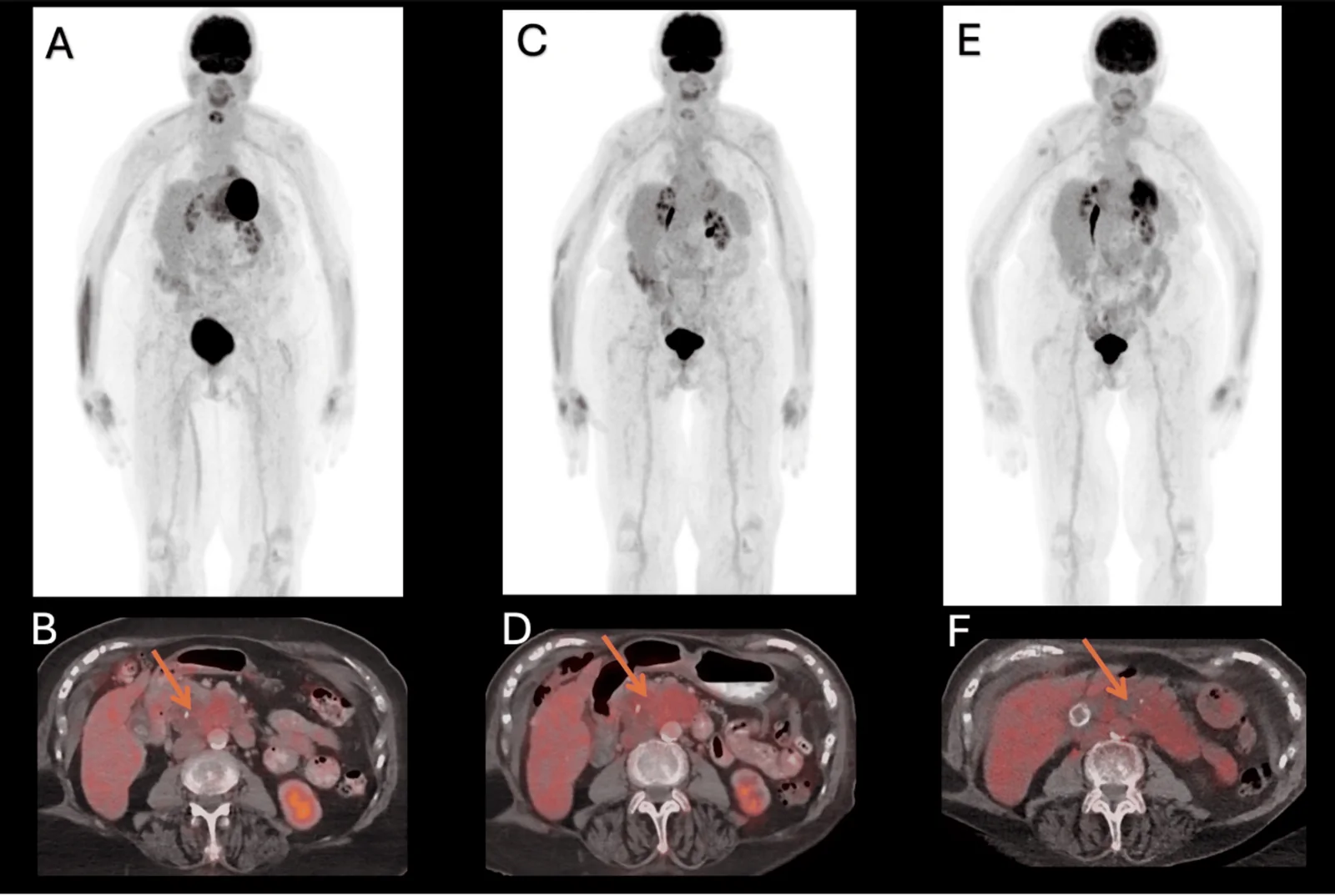
Positron emission tomography-computed tomography (PET/CT) scan images. Baseline scan with whole-body maximum intensity projection (MIP) demonstrating minimal skin thickening of the left thigh without significant FDG uptake prior to resection and otherwise negative for malignancy (A). Selected axial slice demonstrating a mixed solid/cystic, partially calcified mass in the pancreatic head/uncinate process (B, D, F, arrows) with benign cytology on fine-needle aspiration (FNA). Additional scans with similar findings were obtained nine months (C, D) and 15 months (E, F) later (of note, pancreatic findings are slightly changed in appearance due to post-biopsy changes (D, F) and differences in technique (IV contrast diagnostic CT {A-D} and low-dose non-contrast CT {E-F}). Left thigh post-operative changes on subsequent scans (C, E) with no evidence of local recurrence. None of the images demonstrate findings to suggest metastatic disease from Merkel cell carcinoma.

Due to her significant medical comorbidities, including new-onset atrial fibrillation and congestive heart failure, and her inability to tolerate prolonged operative positioning, sentinel lymph node biopsy (SLNB) was deferred. She underwent wide local excision of the left thigh tumor three months after the initial biopsy. Excision pathology confirmed residual MCC tumor measuring approximately 12 mm in greatest dimension. Peripheral margins were uninvolved, with the carcinoma 8 mm from the nearest peripheral margin, and the deep margin was uninvolved at a distance of 15 mm. Lymphovascular invasion was not identified. Stage classification (TNM, AJCC Eighth Edition) was pT1 cN0 cM0 (clinical stage I) [10]. An initial clinical presentation size of 1.8 cm was used for T staging per current guidelines, as the pathologic size at the time of excision represented the remaining tissue after the initial biopsy and possible tissue contraction due to processing. In the absence of SLNB, which is needed for pathologic lymph node staging, a clinical rather than pathologic stage was assigned under the current staging guidelines, acknowledging the residual uncertainty regarding occult nodal disease, as PET/CT is insensitive to nodal micrometastases. 

In conjunction with both medical and surgical oncology, and after discussion with the patient regarding her preferences, the decision was made to forego adjuvant systemic therapy and radiotherapy given the patient’s significant acute comorbidities. Clinical surveillance was recommended via serial PET/CT scans, particularly as serial imaging was already indicated for surveillance of the incidental pancreatic lesion. Nine- and 15-month follow-up PET/CT scans showed no evidence of recurrence, and the pancreatic lesion remained grossly unchanged (Figures 3A-3F). At her most recent follow-up, 16 months after excision, she showed no clinical evidence of recurrence.

## Discussion

Merkel cell carcinoma (MCC) is a highly aggressive skin cancer and the second leading cause of skin cancer death after melanoma [11,12]. Despite advancements in treatment, the five-year disease-specific survival remains around 64%, underscoring the unmet need for novel therapies [12]. MCC predominantly affects the elderly, with a median age of diagnosis between 75 and 79 years, and occurs more frequently in men (male-to-female ratio of 2:1) [11-13]. The White population has a 25-times greater risk of MCC, particularly on the sun-exposed head and neck [13]. While immunosuppression is present in only about 10% of cases, it is associated with a markedly worse prognosis and can drive the disease in younger cohorts [14].

The unusual presentation of MCC as an erythematous patch on a normally non-sun-exposed area (the anterior thigh), with an associated two-month diagnostic delay, demonstrates the importance of biopsying cutaneous lesions that are atypical or non-responsive to standard topical treatments [9]. Histopathological diagnosis of MCC can be challenging. MCC must be distinguished from other small round blue cell tumors, including some aggressive B-cell lymphomas and small cell lung cancer, among others, requiring careful use of immunohistochemical (IHC) markers [3,15,16]. MCC is defined by positivity for neuroendocrine markers (chromogranin A, synaptophysin, CD56) and characteristically shows a perinuclear dot-like pattern for cytokeratin 20 (CK20), while typically being negative for thyroid transcription factor (TTF)-1 (which helps rule out metastatic small cell lung cancer) [3,16].

Early diagnosis of MCC is critical due to its high rate of regional and distant metastasis [8]. Management is stage-dependent. For clinically node-negative localized disease, wide local excision with sentinel lymph node biopsy is central to management, with postoperative radiotherapy to the tumor bed and, in selected cases, the nodal basin, guided by recurrence risk, pathologic findings, and patient factors [17]. Systemic therapy, including immune checkpoint inhibition, is established primarily in unresectable or metastatic disease, with perioperative and adjuvant approaches still evolving [17]. Our patient’s staging workup revealed a concurrent pancreatic lesion with benign cytology and subsequent imaging stability that was negative for metastatic spread; however, it was complicated by several new medical conditions, highlighting the necessity of tailoring standard-of-care treatments in elderly patients burdened by acute, severe medical comorbidities.

## Conclusions

This case illustrates an atypical presentation of Merkel cell carcinoma presenting as an erythematous patch in a non-sun-exposed area. It reinforces a critical dermatologic pearl - clinicians should maintain a high index of suspicion and have a low threshold to biopsy persistent, growing, solitary, or otherwise atypical lesions and rashes in which the clinical course raises diagnostic uncertainty. Accurate diagnosis of MCC from biopsy tissue relies upon its distinct immunohistochemical profiling. Finally, multidisciplinary MCC management is guided by current stage-based clinical guidelines and the individual patient’s physiological status and comorbidities, which may at times limit a complete workup, including, in this case, pathological nodal evaluation.
